# Health promotion for children, mothers and families: here’s why we should “think about it before conception”

**DOI:** 10.1186/1824-7288-39-68

**Published:** 2013-10-25

**Authors:** Carlo Corchia, Pierpaolo Mastroiacovo

**Affiliations:** 1 ICBD, Alessandra Lisi International Centre on Birth Defects and Prematurity, Rome, Italy

**Keywords:** Preconception health, Women’s health, Prematurity, Birth defects, Risk factors, Reproduction, Primary prevention

## Abstract

About 90,000 preterm infants or babies with birth defects are born in Italy every year, nearly 250 per day. These congenital conditions and their outcomes represent the most important burden of disease affecting the health status and the quality of life during infancy, childhood and beyond. In many cases they are fostered by the presence of maternal and/or paternal preconception risk factors whose effects can be modified by primary prevention.

In the contemporary vision of maternal and child health, the traditional gap between preconception period and pregnancy is overcome through promotion of reproductive health and wellness in women, men and couples, regardless of their reproductive plans and possible future pregnancies. This paradigm should become the basic foundation to improve and protect infants’, children’s and adolescents’ health. Useful interventions belong to four broad areas: a) promotion of women’s and couples’ health in general, and protection from risk of adverse events in future pregnancies (if any); b) identification and treatment of conditions that increase the risk of adverse reproductive outcomes; c) help for women (couples) planning for pregnancy; d) identification of genetic risks, and help for independent and responsible decision making.

Pediatricians and neonatologists can effectively promote primary prevention in the interconception period, when parents seek consultation for their previous child, in adolescent medicine, in family health education, in socio-sanitary network, and in advocacy activities in favor of infants and children. These actions should be part of an operational framework including perinatal outreach programs, information campaigns, and focus on problems of high-risk women, children and families.

## Background

The promotion of women’s and couples’ health before conception of their children should become the first-line essential task of the National Health Service if we want to improve future generations’ health.

There are at least three good reasons supporting this statement:

1. About 90,000 preterm infants or babies with birth defects are born in Italy every year, nearly 250 per day [1]; these congenital conditions and their outcomes later in life represent the most important burden of disease affecting the health status and quality of life during infancy, childhood and beyond; in many cases they are fostered by the presence of maternal and/or paternal preconception risk factors whose effects can be modified by primary prevention actions [2];

2. The prevalence of modifiable preconception risk factors is much higher than expected, and there is almost no couple of parents-to-be without at least one;

3. Many babies can be born without severe congenital conditions following interventions aimed at modifying the exposure to potential risk factors before conception.

Primary prevention in the preconception period is therefore the basic mainstay of any program pointing at promotion and protection of infants’, children’s and adolescents’ health [3].

## Main text

### Adverse reproductive outcomes

Adverse reproductive outcomes are numerous and include both pathological and potentially pathological events of reproduction [4]. They may be grouped in three main categories: a) those in relation with the couples’ reproductive activity (e.g., infertility and subfertility, spontaneous abortions, unwanted or untimed pregnancy, pregnancy termination for psycho-social reasons); b) pregnancy complications (e.g., gestational diabetes, ectopic pregnancy, pre-eclampsia, placental abruption, premature rupture of membranes); c) those pertaining to fetus/newborn (e.g., congenital malformations, intra-uterine growth restriction, congenital disabilities – motor, sensorial, cognitive, behavioral –, congenital tumors, SIDS, prematurity, chromosomal and monogenic genetic diseases).

Adverse reproductive outcomes have been usually interpreted as possible consequences of many relatively rare, largely behavioral, risk factors. This approach has generated what has been defined a “fragmented epidemiology”, that in turn has been responsible for fragmented health policies and programs [5]. In particular, in the old paradigm of maternal and child health the preconception period was largely neglected and separated from events occurring and decision made after pregnancy diagnosis. In the new paradigm this gap between preconception period and pregnancy is overcome through promotion of reproductive health. In more general terms this means promotion of health and wellness of women, men and couples, regardless of their reproductive plans and possible future pregnancies, along with attention for prenatal, perinatal and post-natal periods, and beyond. In other words, throughout their whole lifespan [6].

It is far-back known, although often overlooked, that the biological being starts at conception. This statement is undeniable, independently of any ethical debate about the concepts of life or person. The moment of conception and the earliest steps of embryo development have been recognized as essential for humankind wellness. Epigenetic reprogramming occurs soon after conception [7]. Placenta and embryo organs develop immediately, in the following days. On an average, women schedule their first prenatal visit 3–4 weeks after pregnancy has been diagnosed; that is, too late to start with a comprehensive and effective program of primary prevention of the adverse reproductive outcomes and congenital conditions. It is therefore critical to “think about it before conception” and to maintain optimal health during the entire reproductive life and well before the beginning of pregnancy [8]. This attitude would allow scheduling the number and spacing of pregnancies, and to getting ready to have a healthy baby. So far the prevalent interest of health care providers has been pointing to future mothers (“M”); now the “M” should be turned upside down and become “W” for women, without, however, excluding fathers and males as well [6]. Actually, women’s (and men’s) health in their reproductive age is neither as good as it is usually believed, nor as good as it could be. Moreover, the advantages derived from the control of known risk factors are not only limited to the adverse outcomes of reproduction, but can be extended up to the adult and old age, both for parents and their offspring [9].

### A “road map” for pediatricians and pediatrics

The cultural and professional domain of pediatrics should include the primary prevention of the adverse reproductive outcomes. If it is agreed upon that health should be promoted “any time any healthcare provider sees a reproductive age woman” [4,10], then it is undeniable the relevant role of pediatricians and neonatologists [11,12]. This commitment is to be regarded as inherent to and not an extension of their usual professional role. In Italy, nearly 50% of babies are born to couples with previous children, and virtually all mothers come in contact with pediatricians/neonatologists. During consultation, counseling and some simple instruments for self-evaluation and information are among tools pediatricians can use to promote preconception health, especially in the interconception period [8,13].

Although the effectiveness of women’s health promotion carried out by pediatricians in the interconception period has not been evaluated in formal studies, a good amount of evidence is available about the usefulness of some core preventive interventions in the pre-pregnancy period [10,14]; this evidence is the basis for extensive recommendations to improve preconception health and health care [15]. Pediatricians have also an important role in adolescent medicine, in family health education, in socio-sanitary networks, and in advocacy actions in favor of infants and children. All these activities can be carried out by pediatricians with great competence and proficiency.

Useful interventions for the promotion of reproductive health can be grouped in four broad areas:

A. Promotion of women’s and couples’ health in general, and protection from risk of adverse events in future pregnancies (if any);

B. Identification and treatment of conditions that increase the risk of adverse reproductive outcomes;

C. Help for women (couple) planning for pregnancy, in order to face and properly manage the risk factors of adverse reproductive outcomes;

D. Identification of genetic risks, and help to make responsible decisions.

Each of these areas includes a series of specific activities. For example, in area A we can found: vaccinations against rubella, chickenpox, tetanus, whooping cough; sex education; individual counseling for fertility control; prevention of pregnancies before the age of 20 and information about risks associated with advanced reproductive age; prevention of short interpregnancy intervals; promotion of healthy lifestyle; identification of and action for women/couples with socio-economic difficulties, stress, depression, domestic violence; active advice for daily supplementation with 0.4 mg folic acid. Other examples could be given for the remaining three areas [4,16].

Pediatricians can represent an important source of advice during every working circumstances: as family pediatricians, in community health centers, in private offices, in health authorities’ districts, in hospitals’ inpatient and outpatient departments. Possible barriers however exist; they include concern about reaction of mothers, inadequate knowledge of possibilities to assist them, and financial obstacles [11]. These drawbacks can be overcome a) by fostering the development of a positive attitude towards health promotion, and b) by including preconception and interconception prevention in the university and post-graduate/post-doctoral curricula as well as in the continuous medical education programs.

## Discussion

Two aspects must be highlighted. The first one, that has already been hinted at, implies that risk factors (environmental, biological and behavioral) may occur throughout almost a woman's entire life, in particular from her own conception to the birth of her children, a fact that partly explains the observed differences in health. Many generations might therefore be needed in order to have a disease-burden reduction as a consequence of effective prevention interventions. The second aspect is linked to the need of identifying the most appropriate actions for minority high-risk population groups. In this respect, the so called “vertical” (non-integrated) programs have not produced substantial and long-acting results; they are characterized by distinct short- or medium-term targets, and are based on activities financed separately and running in parallel with those normally offered by the National Health Service. On the other hand, the so called “horizontal” (integrated) programs may represent a valid and more effective option. They can be focused on integrated interventions for interrelated health problems, running in services dedicated to prevention and primary health care, and routinely financed by the National Health Service with no or only small additional funding [17]. Within an integrated program it is also possible to fulfill “adaptive” preventive actions, modulated in relation to the needs, awareness, attitudes and preferences of single people and population groups [18].

## Conclusions

A unique conceptual framework is needed to promote women’s and couples’ health (reproductive health). Perinatal outreach programs, information campaigns to increase the awareness of people about health gain and preservation, and focusing on problems of high-risk women, children and families, should all become part of such a comprehensive framework.

## Competing interests

The authors declare that they have no competing interests.

## Authors’ contribution

Both CC and PM have made substantial contributions to conception and design of the paper, have been involved in drafting the manuscript and revising it critically for important intellectual content, and have given final approval of the version to be published.

## Authors’ information

Carlo Corchia: Consultant neonatologist and epidemiologist; deputy Director, Alessandra Lisi International Centre on Birth Defects and Prematurity, WHO collaborating centre, Rome, Italy; former Director, Neonatal Intensive Care Unit, Bambino Gesù Childrens’ Hospital, Rome, Italy.

Pierpaolo Mastroiacovo: Full professor of Pediatrics and epidemiologist; Director, Alessandra Lisi International Centre on Birth Defects and Prematurity, WHO collaborating centre, and Pensiamoci Prima Project, Rome, Italy; Director, Centre of the International Clearinghouse for Birth Defects Surveillance and Research; former Director, Department of Pediatrics, Catholic University, Rome, Italy.
